# Thrombotic Microangiopathy with Complement Factor H Gene Mutations Unassociated with Atypical Hemolytic Uremic Syndrome

**DOI:** 10.4274/tjh.2015.0084

**Published:** 2015-08-01

**Authors:** Yeşim Oymak, Tuba Hilkay Karapınar, Yılmaz Ay, Esin Özcan, Neryal Müminoğlu, Sultan Aydın Köker, Ersin Töret, Afig Berdeli, Erkin Serdaroğlu, Canan Vergin

**Affiliations:** 1 Dr. Behçet Uz Children’s Hospital, Clinic of Hematology, İzmir, Turkey; 2 Ege University Faculty of Medicine, Department of Genetics, İzmir, Turkey; 3 Dr. Behçet Uz Children’s Hospital, Clinic of Nephrology, İzmir, Turkey

**Keywords:** thrombotic microangiopathy, eculizumab, aHUS, CFH gene

## TO THE EDITOR

Atypical hemolytic uremic syndrome (aHUS) is a rare multigenic disorder characterized by thrombotic microangiopathy (TMA). Among the genes that are associated with aHUS, mutations in the complement factor H (CFH) gene are the most common genetic cause of the disease. Several specific gene mutations have been identified in patients with aHUS [1,2,3].

A 5-year-old boy was admitted to our hospital with fatigue. Physical examination revealed pallor and hepatomegaly. His blood pressure was within the normal range. A blood smear showed hemolysis with 10% schistocytes and polychromasia. He had anemia with 5.5 g/dL hemoglobin level and 14.6% reticulocyte level. He had also thrombocytopenia (48,000/mm3) and elevated lactate dehydrogenase (LDH) as 1066 U/L. A direct Coombs test was negative. The other blood parameters of the patient were as follows: haptoglobin 5 mg/dL (reference range: 41-165 mg/dL), C3 122 mg/dL (reference range: 79-152 mg/dL), creatinine level 0.5 mg/dL, blood urea nitrogen 12 mg/dL, and indirect bilirubin 7 mg/dL. Urine analysis was normal. On the second day of admission, the patient’s thrombocyte count dropped to 38,000/mm3, his LDH level remained elevated, and schistocytes were still present on his peripheral blood smear. As for the blood smear and other TMA symptoms, first plasmapheresis was started, which lasted 20 weeks. Then treatment was continued with 600 mg of eculizumab weekly for the first three weeks and followed by once every 2 weeks (total 11 doses). Although the patient had TMA symptoms he didn’t have renal insufficiency. Also ADAMTS-13 activity was 48% (reference range: 40%-130%) and ADAMTS-13 antibody was negative.

Four months after stopping eculizumab, the patient’s levels of hemoglobin, thrombocytes, reticulocytes, haptoglobin and LDH were 11 g/dL, 150,000/mm3, 0.87%, 46.4 mg/dL (reference range: 41-165 mg/dL) and 385 U/L, respectively. Informed consent was obtained for genetic testing and publishing the patient’s data from his parents.

**Figure 1:**
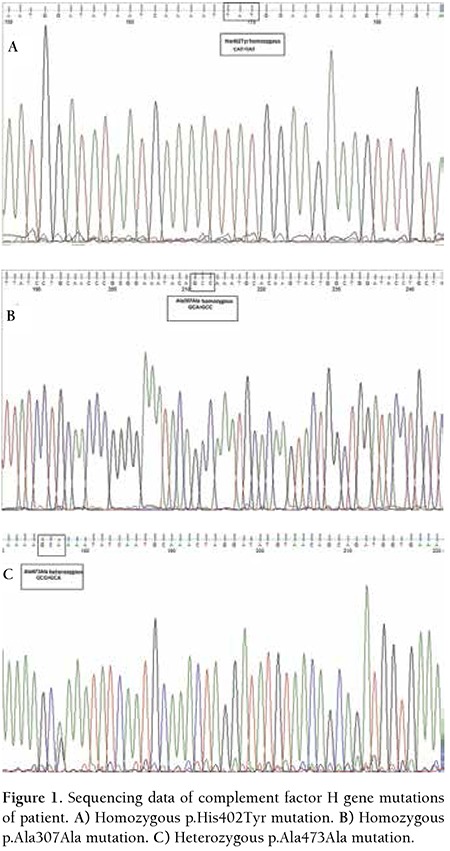
Sequencing data of complement factor H gene mutations of patient. A) Homozygous p.His402Tyr mutation. B) Homozygous p.Ala307Ala mutation. C) Heterozygous p.Ala473Ala mutation.

DNA sequencing analysis of the patient revealed a homozygous p.His402Tyr mutation due to a p.1204 C>T change in exon 9, a homozygous p.Ala307Ala mutation due to a p921A>C change in exon 7 and a heterozygous p.Ala473Ala mutation due to a p.1419G>A change in exon 10 of the CFH gene (Figure 1).

CFH gene mutations are the most commonly observed genetic changes in patients with aHUS and they are responsible in 20%-30% of the patients [1,2,3]. Eighty-seven CFH gene mutations associated with aHUS have been described to date [4,5]. The CFH gene mutation that we identified in our case has been associated with membranoproliferative glomerulonephritis (MPGN) and age-related macular degeneration (AMD) [6,7,8]. The location of the p.His402Tyr mutation in the functional domain of the protein suggests that it might have a pathogenic effect in patients with aHUS [5].

In conclusion CFH gene analysis was performed to confirm whether the patient had aHUS or not. However, we have found CFH gene mutations that are not specific for aHUS. Epigenetic factors might have triggered the patient’s phenotype. Also p.His402Tyr mutation may cause TMA with a milder clinic feature than that of other aHUS specific CFH gene mutations.

**Conflict of Interest Statement**

The authors of this paper have no conflicts of interest, including specific financial interests, relationships, and/or affliations relevant to the subject matter or materials included.
